# Relationship between the level of mixed chemicals in male urine and the prevalence of male cancers, especially prostate cancer

**DOI:** 10.3389/fpubh.2025.1544174

**Published:** 2025-03-12

**Authors:** Bin Zhang, Hao Sun, Bin Zhu, Mengmeng Wang, Bingli Zuo, Jiuming Dai

**Affiliations:** ^1^Binhai County People's Hospital, Yancheng, Jiangsu, China; ^2^Qingdao Hospital, University of Health and Rehabilitation Sciences (Qingdao Municipal Hospital), Qingdao, China; ^3^Licang District Geriatric Hospital, Qingdao, China; ^4^Xiyuan Hospital, Chinese Academy of Traditional Chinese Medicine, Beijing, China; ^5^Shandong Cancer Hospital and Institute, Shandong First Medical University and Shandong Academy of Medical Sciences, Jinan, China

**Keywords:** cancers, NHANES, men, prostate, mixed chemicals, urine

## Abstract

**Objectives:**

The aim of this study was to investigate the relationship between mixed chemicals in urine and the prevalence of cancers in men.

**Methods:**

A total of 1,068 male subjects were included in this study. Analyses were performed by several analytical methods to ensure the stability of the results: one-way analysis, WQS analysis, Qgcomp analysis, BKMR analysis, and Restricted Cubic Spline (RCS).

**Results:**

In the final adjusted model, each 1 increase in ln-transformed BPS increased the risk of developing cancerous prostate by 49% (95% CI: 1.00–2.20). The results of multiple sensitivity analyses by WQS and Qgcomp showed that the mixed chemicals was positively correlated with the prevalence of cancers and prostate cancer in men. In the final adjusted model, each quartile increase in the WQS index was associated with a 78% (OR: 1.78, 95% CI: 1.10–2.87) increase in the risk of cancers and a 148% (OR: 2.48, 95% CI: 1.07–5.71) increase in the risk of prostate cancer. Each quartile increase in the Qgcomp index was associated with a 59% (OR: 1.59, 95% CI: 1.09–2.33) increase in the risk of cancers, and a 105% (OR: 2.05, 95% CI: 1.04–4.06) increase in the risk of prostate cancer.

**Conclusion:**

In conclusion, this study showed a positive correlation between the concentrations of the three groups of mixed chemicals in urine and the prevalence of cancers in men, as well as a positive correlation with the prevalence of prostate cancer.

## 1 Introduction

The metabolite composition of urine is closely related to changes in the metabolic products of the organism and reflects the need for solute and water balance in the body. Urine is considered a source of biomarkers that can reflect the health of the human organism (1). Urinalysis is one of the oldest medical tests including physical, chemical and microscopic. Urinalysis is the best candidate for continuous health monitoring due to its inherent metabolic phenotypic individualisation, quantitative nature, non-invasive nature, and the ability to detect multiple analytes (2). A clinical study stated that about 4,500 metabolites have been identified in urine. They are strongly associated with about 600 human diseases including, but not limited to, cancer, neurological diseases, and infectious diseases (3). Studies have shown that differences in the composition of urine in healthy and diseased populations are important to study for the prevention and treatment of cancers such as bladder, prostate, and thyroid cancers (4–6).

Bisphenol A (BPA) is a chemical with endocrine disrupting properties, and with the increase in consumption by the population, alternatives to bisphenol have emerged, especially bisphenol S (BPS) (6). Both BPA and BPS have now been detected in urine in large quantities (7). Studies have shown multiple associations between urinary levels of BPA and BPS and the expression of oxidative stress markers (8). A related study found a positive correlation between urinary concentrations of BPA and its analogs and male sex hormone levels (9). In addition, BPA and BPS are strongly associated with thyroid function, obesity, breast cancer, and depression (10–13). Parabens (PB) are widely used as antimicrobial and preservative agents. However, more and more studies have shown that parabens, as endocrine-disrupting chemicals, have important effects on human health (14). According to the Centers for Disease Control and Prevention (CDC), it was found that both methylparaben (MPB) and propylparaben (PPB) were detected in urine samples from more than 96% of the US population (15). PB exposure was found to have a potential regulatory role in prognostic gene expression in prostate cancer (16). More importantly, PB has a significant impact on the migration of a wide range of cancer cells (17). Dichlorophenol (DCP) is an endocrine disruptor commonly found in consumer and industrial products. 2,4-DCP and 2,5-DCP were detected in more than 81% of urine samples collected by the National Health and Nutrition Examination Survey (NHANES) (18). There is a positive correlation between the concentration of DCP in urine and the development of metabolic syndrome (19). In another study on the concentration of DCP in urine and diabetes mellitus demonstrated a potential association between the concentration of DCP in urine and diabetes mellitus in adults (20). In addition, DCP has an important effect on androgen induction in prostate cancer cells (21). It was found that studies of the relationship between the above mixture of chemicals with common characteristics and cancer risk in men are relatively rare. Therefore, the present study explored the relationship between mixed chemicals and the risk of cancer in men.

Prostate cancer is the second most common male cancer and the fifth leading cause of cancer death (22). Data analyses have shown that the prevalence of prostate cancer is steadily increasing globally, and the mortality rate is also increasing year by year (23, 24). Although the relationship between urinary metabolite composition and numerous diseases is becoming more widely studied. No study has yet analyzed the association between urinary metabolite composition in men and pan-cancer in men, particularly prostate cancer. Therefore, it is important to explore the association between urinary metabolite composition and cancers in men for early prevention, screening, and diagnosis of cancers.

In this study, six common urinary metabolite components (BPA, BPS, MPB, PPB, 2,4-DCP, and 2,5-DCP) were analyzed in a comprehensive measurement using NHANES data from 2013 to 2016. The association between the levels of these chemicals and pan-cancer in men, particularly prostate cancer, has been investigated by a variety of statistical methods. The results of the study showed a positive correlation between the level of chemicals in urine and pan-cancer in men. Among them, MPB had the highest weight of positive effect on cancer prevalence and BPS had the highest weight of positive effect on prostate cancer. This study also verified the stability of the results through a variety of statistical analyses. This study evaluates the association between chemicals in male urine and pan-cancer risk in men. It aims to provide some potential biological markers for early screening and early diagnosis of cancer in men.

## 2 Materials and methods

### 2.1 Study population

NHANES is a stratified, multi-stage periodic survey conducted by the CDC. The sample survey data is a trustworthy nationally representative sample study. The study followed ethical and moral standards and norms. The study was approved by the Ethical Review Board of the National Center for Health Statistics (NCHS) and written informed consent was also obtained from the participants. Data for the study were obtained by randomly selecting participants and conducting interviews, physical examinations, etc., at regular or irregular intervals. Detailed information is available at http://www.cdc.gov/nchs/nhanes/index. All participants in this experiment were screened from the 2013–2016 NHANES database.

### 2.2 Measurement of chemicals in urine

On-site urine samples are collected at the Mobile Examination Center (MEC). Samples were stored at −20°C until analyses were performed by the Laboratory Sciences Department of the Organic Analytical Toxicology Branch, National Center for Environmental Health (Atlanta, Georgia). Phenol, parabens, and chlorophenols were included in this study because they were detected above the detection limit in at least 90% of the participants. Concentrations of BPA, BPS, MPB, PPB, 2,4-DCP and 2,5-DCP were measured by on-line solid-phase extraction combined with high-performance liquid chromatography and tandem mass spectrometry (on-line SPE-HPLC-isotope dilution-MS/MS). Detailed information on chemical measurement methods is available from NHANES Laboratory Methods.^1^ For analytical results below the lower limit of detection, this study divided the lower limit of detection by the square root of two.

### 2.3 Covariate

A standardized questionnaire and MEC were used to collect information about participants’ age, gender (male, female), race (Mexican American, other Hispanic, non-Hispanic white, non-Hispanic black, and other races), educational attainment (less than high school, high school and more than high school), marital status (married / living with partner, widowed / divorced / separated, never married), smoking behavior (never, former, and now), alcohol consumption(never, former, mild, moderate, heavy), diabetes (yes and no), hypertension (yes and no), family property income ratio (PIR), body mass index (BMI), total energy intake, urea creatinine (UCR), urea nitrogen (UBU), alanine aminotransferase (ALT), aspartate aminotransferase (AST), and uric acid (UA). Alcohol consumption is divided into five levels: 1.never (drinked less than 1 drinks for females, 2 drinks for males in the past months), 2.former (drinked more than 1 drinks for females), 3mild (≥1 drinks per day for females), 4.moderate (≥2 drinks per day for females), 5.heavy (≥3 drinks per day for females). Diseases of the body systems and organs are confirmed by a doctor or health professional.

### 2.4 Statistical analysis

The following statistical methods were used: (1) Univariate analyses, with separate models and adjusted analyses for each chemical in the urine. (2) WQS and qgcomp analyses, to identify significant chemicals and to estimate mixing effects for all metals. (3) BKMR analyses, to model individual and combined effects of mixtures as well as non-linear relationships and interactions. (4) Restricted cubic spline (RCS), to explore the non-linear relationship between each chemical and cancer risk.

**Figure 1:**
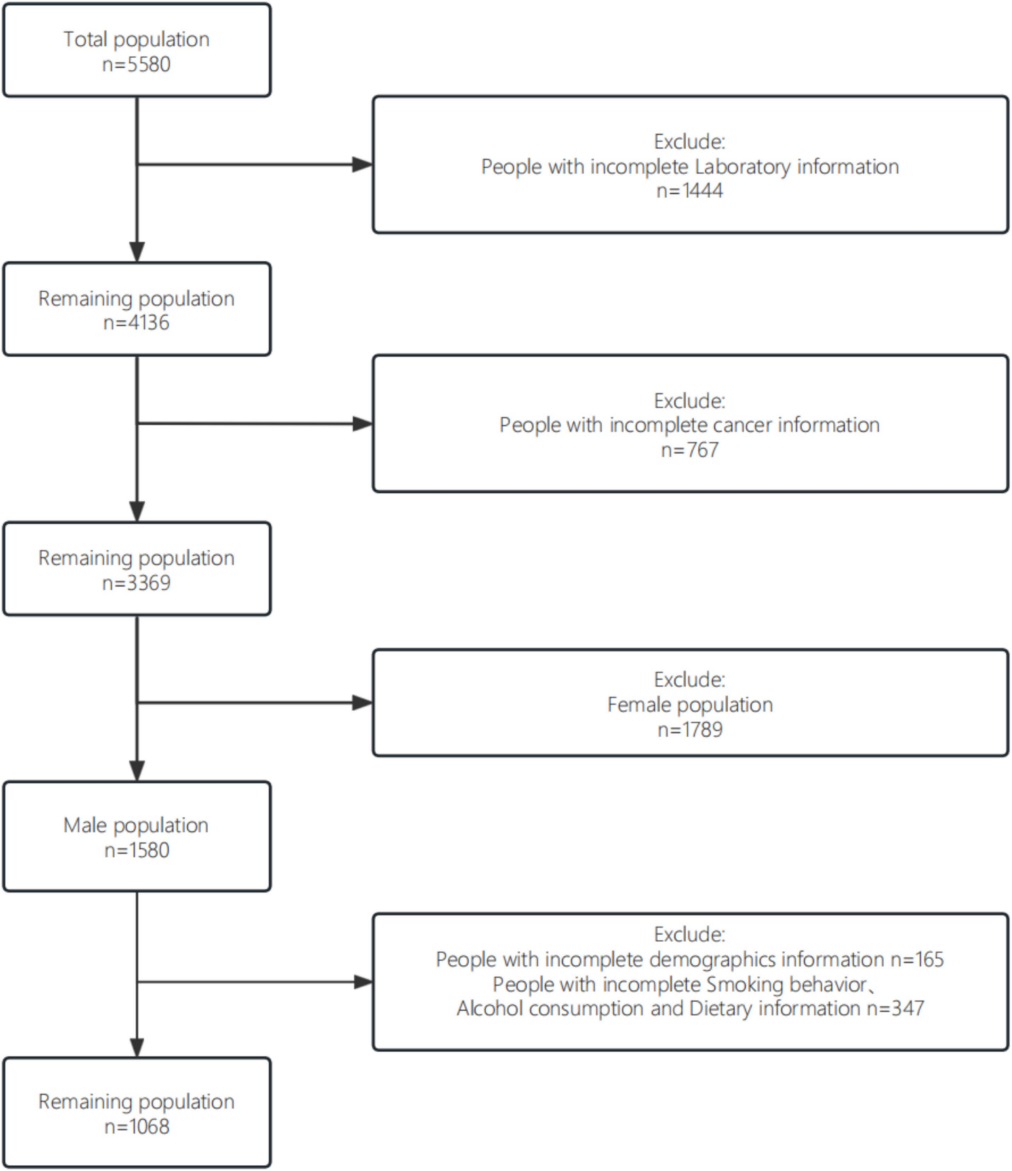
Flowchart of the study design and sampling.

The study design, sampling, and exclusion process was shown in Figure 1. For random missing samples, the study used multiple interpolation to fill in. For non-random missing data, they were excluded from this study to maximize the representativeness of the sample. Filtered data were analyzed using R (v.4.2.1). Measures in the demographic characteristics data were described as mean (SD) to describe the distribution, and t-tests were used to determine differences between groups. Count data were expressed as N (%) and the chi-square test was used to determine differences between groups. Due to the severe right skewed distribution of chemicals in urine, all six chemicals as continuous variables in the analysis were in transformed to improve the normal distribution.

In the regression analyses, this study first used multiple logistic regression for continuity analysis. Due to the complex multi-stage sampling design of NHANES, this study calculated weights and performed survey-weighted multivariate logistic regression to assess associations between individual chemicals and pan-cancers and prostate cancer in men. In addition, this study performed sensitivity analyses adjusted for age, race, education level, marital status, PIR, BMI, smoking, alcohol consumption, hypertension, diabetes, energy intake, UCR, UBU, ALT, AST, and UA levels.

WQS regression was employed to assess the mixture effect of six chemicals and to identify the chemical that most likely drives the association between the mixture and the prevalence of pan-cancers and prostate cancer in men. The WQS model assumes that the mixture components have a unidirectional effect (either all positive or all negative) on the outcome, which is a key assumption of this method. To implement the WQS regression, the concentrations of the six chemicals were first divided into quartiles and combined into an index. The weights for each chemical were empirically derived through bootstrap sampling, and the final WQS index was calculated to represent the mixed effect of the six chemicals (25).

Specifically, we assumed positive coefficients for each component, as prostate cancer and other male cancers are hypothesized to be positively associated with chemical exposure. The dataset was split into a test set (40% of the total samples) and a validation set (60% of the total samples) to ensure robustness. We performed 1,000 bootstrap iterations to estimate the weights and the WQS index. The odds ratio (OR) with a 95% confidence interval (CI) was interpreted as the combined effect of each quartile increase in the mixed chemical exposure on the outcome.

To validate the reliability of the results, sensitivity analyses were conducted by constructing three adjusted models. These models were adjusted for potential confounders, including age, race, education level, marital status, poverty-to-income ratio (PIR), body mass index (BMI), smoking status, alcohol consumption, hypertension, diabetes mellitus, energy intake, urinary creatinine (UCR), urinary urea nitrogen (UBU), alanine aminotransferase (ALT), aspartate aminotransferase (AST), and uric acid (UA) levels.

In addition to WQS regression, we applied the QGC model to explore the relationship between co-exposure to the six chemical mixtures and cancer prevalence in men. Unlike WQS, the QGC model does not assume directional homogeneity (i.e., it allows for both positive and negative weights for individual components in the mixture). This flexibility makes QGC particularly useful for identifying chemicals with opposing effects on the outcome.

The QGC model estimates the combined effect of the mixture by calculating weights for each chemical component based on their quantized exposure levels. Similar to the WQS approach, the chemical concentrations were divided into quartiles, and the model was run with 1,000 bootstrap iterations to ensure stable estimates. The number of iterations was chosen based on previous literature and computational feasibility. The QGC model also adjusted for the same set of confounders as the WQS regression to ensure comparability between the two methods (26).

To enhance the robustness of our findings, we constructed three models for sensitivity analyses in both the WQS and QGC frameworks. These models were designed to test the stability of the results under different assumptions and adjustments. For reproducibility, we set a random seed (seed = 3) before running the bootstrap iterations in both WQS and QGC analyses.

Finally, this study was evaluated using a mixture analysis model (BKMR) for further analysis. Individual, combined effects of six chemicals on pan-cancers and prostate cancer in men were flexibly estimated using Gaussian kernel functions through a Bayesian statistical learning approach. Notably, BKMR is capable of identifying interactions and non-linear relationships of mixture components. Based on the Pearson correlation coefficients and their similar sources of exposure, BPS and BPA were grouped as the first group, MPB and PPB as the second group, and 2,4-DCP and 2,5-DCP as the third group.


$$Y_{i}=h\left[\begin{array}{l} \mathrm{Grou}p_{1}=\left(\mathrm{BPS,BPA}\right),\mathrm{Grou}p_{2}=\left(\mathrm{MPB,PPB}\right),\\ \mathrm{Grou}p_{3}=\left(2.4-\mathrm{DCP,}2.5-DCP\right) \end{array}\right]+\beta Z_{i}+e_{i}$$

Where Yi denoted the outcome, Zi denoted the covariates, and β denoted the corresponding regression coefficients, respectively. h() was an exposure-response function based on non-linearity and or interactions between mixture components. This study used a hierarchical variable selection procedure with 20,000 iterations through a Markov chain Monte Carlo algorithm. Meanwhile, group posterior inclusion representing the probability of mixing groups were calculated from the BKMR model to determine the relative importance of each exposure to the outcome. This study also assessed the association between changes in single chemical concentrations and cancer prevalence in men when other chemicals were fixed at different percentile concentrations. Bivariate exposure-response functions were used to explore potential interactions between different chemicals. All 16 covariates were adjusted for in the model.

Finally, this study used RCS to explore the dose–response relationship of phenol, parabens and chlorophenols on pan-cancers and prostate cancer risk in men. It has the flexibility to explore the non-linear relationships involved. This study used the median value for each chemical as a reference point and adjusted for all 16 covariates. All analyses were performed using R software (4.2.1). All statistical analyses were performed using the software packages base, qWQS, Qgcomp, BKMR, rms, stats, and survey. *p* values less than 0.05 were considered statistically significant.

## 3 Results

The general characteristics of the study subjects were shown in Table 1. A total of 1,068 male subjects were included and the number of cancer was 113, which was 10.58% of the total study population. The number of prostate cancer was 32 with a prevalence of 3.00 per cent. Age, race, marital status, PIR, smoking, alcohol consumption, hypertension, diabetes mellitus, and UCA levels were significantly different between the two groups.

**Table 1:** Basic characteristics of the study population.

Characteristics	Cancer		*p*-value
	No	Yes	
Total	955(89.42)	113(10.58)	
Age – years	45.78(0.90)	65.49(1.46)	<0.01
Race–%			<0.01
Non-Hispanic Black	208(10.65)	18(3.97)	
Non-Hispanic White	374(66.82)	83(91.24)	
Mexican American	143(8.79)	2(0.50)	
Other Hispanic	105(6.40)	8(1.42)	
Other Race	125(7.35)	2(2.86)	
Education level – %			0.11
Less than high school	202(14.36)	10(6.49)	
High school	235(22.98)	31(20.90)	
More than high school	518(62.65)	72(72.61)	
Marital Status–%			<0.01
Married/living with partner	634(69.63)	82(80.20)	
Widowed/Divorced/Separated	134(10.79)	24(15.20)	
Never married	187(19.58)	7(4.60)	
Family PIR – %	3.10(0.10)	3.89(0.16)	<0.01
BMI – kg/m2	29.44(0.28)	30.31(0.92)	0.35
Smoking behavior–%			<0.01
Never	488(53.66)	40(36.12)	
Former	250(25.99)	62(54.03)	
Now	217(20.35)	11(9.85)	
Alcohol consumption–%			<0.01
Never	82(7.29)	5(1.48)	
Former	183(15.49)	21(14.26)	
Mild	366(41.08)	72(71.98)	
Moderate	111(14.10)	10(7.40)	
Heavy	213(22.03)	5(4.88)	
Energy–kcal	2428.11(37.55)	2271.17(107.28)	0.18
Diabetes–%			0.09
No	778(85.92)	82(77.77)	
Yes	177(14.08)	31(22.23)	
Hypertension–%			<0.01
No	566(62.18)	36(39.49)	
Yes	389(37.82)	77(60.51)	
UCR – mg/dl	0.99(0.01)	1.03(0.02)	0.13
UBU – mg/dl	14.55(0.24)	17.50(0.80)	<0.01
ALT–U/L	30.23(0.83)	24.33(0.81)	<0.01
AST – U/L	27.58(0.60)	25.79(0.90)	0.13
UA – mg/dl	6.06(0.07)	6.15(0.18)	0.60

The distribution of the six chemicals was shown in Table 2. The 5th, 25th, 50th, 75th and 95th quartiles were described and his arithmetic mean and detection rate were calculated. Of the six chemicals, the most abundant was MPB and the least abundant was BPS.

**Table 2:** Distribution of chemicals in male urine.

Chemicals	Detection frequency	LOD	GM	Mean	Selected percentiles				
					5th	25th	50th	75th	95th
BPA – ng/ml	0.9539	0.2(μg/L)	1.33	2.46	0.20	0.60	1.30	2.70	7.80
BPS – ng/ml	0.9012	0.1(μg/L)	0.54	1.61	0.07	0.20	0.50	1.10	5.00
MPB – ng/ml	0.9867	1.0(μg/L)	29.43	141.95	2.40	9.00	24.25	85.93	698.10
PPB – ng/ml	0.9861	0.1(μg/L)	2.79	33.08	0.20	0.60	1.80	11.60	154.16
2,4-DCP – ng/ml	0.9368	0.1(μg/L)	0.73	4.59	0.07	0.30	0.60	1.50	10.50
2,5-DCP – ng/ml	0.9784	0.1(μg/L)	4.30	147.66	0.24	1.00	2.90	12.70	297.48

### 3.1 Correlation analysis of chemicals

**Figure 2:**
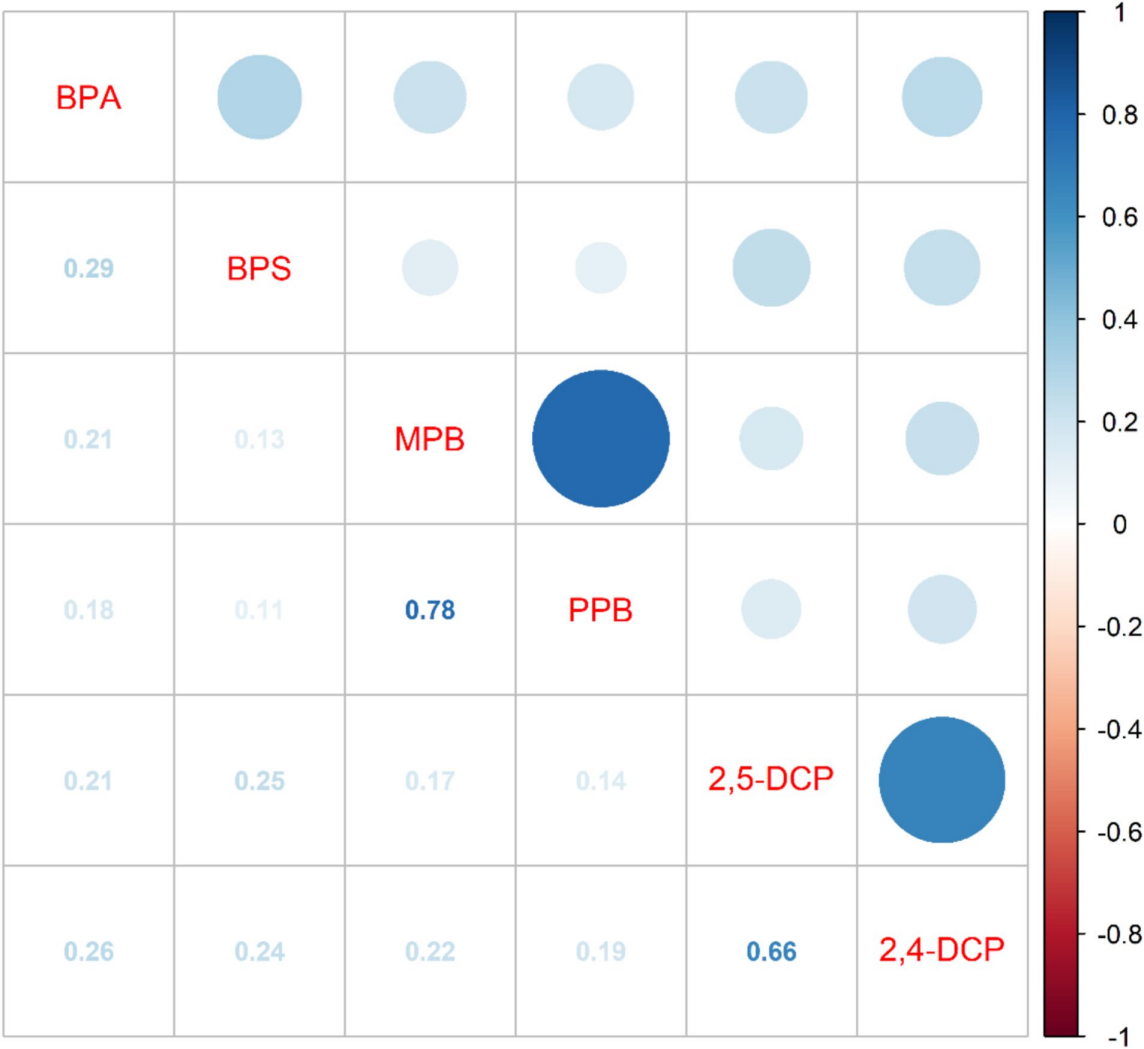
Results of the correlation analysis between the six chemicals.

The correlation analysis between the six chemicals was shown in Figure 2. The intergroup correlations for all three groups of chemicals were greater than the correlations with the other groups. The strongest correlation was between MPB and PPB (*r* = 0.78). This was followed by 2,4-DCP and 2,5-DCP (*r* = 0.66).The weakest correlation was between BPA and BPS (*r* = 0.29).

### 3.2 Univariate analysis

The results of the univariate analysis of the association between chemical levels and the prevalence of cancers and prostate cancer in men were shown in Table 3. BPS was positively associated with prostate cancer prevalence both before and after adjustment. In the final adjusted model, each 1 increase in ln-transformed BPS increased the risk of developing cancerous prostate by 49% (95% CI: 1.00–2.20). BPA, BPS, and PPB were negatively, but not significantly, associated with cancer prevalence. MPB, 2,4-DCP and 2,5-DCP were positively associated with cancer prevalence, again without significant differences. BPA and MPB were negatively associated with prostate cancer prevalence, but there were no significant differences. PPB, 2,4-DCP and 2,5-DCP were positively associated with cancer prevalence, again with no significant differences.

**Table 3:** Results of univariate analysis of the association between chemical levels and the prevalence of cancers and prostate cancer in men.

Chemicals		Cancer		Prostate	
		OR(95%CI)	*p*	OR(95%CI)	*p*
BPA	Model 1	0.97(0.74,1.26)	0.80	0.96(0.57,1.63)	0.88
	Model 2	0.97(0.72,1.29)	0.79	0.95(0.52,1.73)	0.85
	Model 3	0.96(0.66,1.38)	0.75	0.95(0.47,1.93)	0.85
BPS	Model 1	1.00(0.84,1.19)	0.99	1.37(1.07,1.75)	**0.02**
	Model 2	0.98(0.79,1.21)	0.83	1.41(1.04,1.93)	**0.03**
	Model 3	0.97(0.74,1.27)	0.78	1.49(1.00,2.20)	**0.05**
MPB	Model 1	1.09(0.87,1.35)	0.45	0.99(0.81,1.22)	0.96
	Model 2	1.06(0.82,1.38)	0.62	0.95(0.70,1.29)	0.70
	Model 3	1.07(0.78,1.47)	0.59	0.96(0.68,1.34)	0.74
PPB	Model 1	1.00(0.86,1.17)	0.95	1.14(0.99,1.30)	0.07
	Model 2	0.97(0.80,1.18)	0.74	1.05(0.88,1.26)	0.53
	Model 3	0.98(0.79,1.23)	0.85	1.09(0.89,1.34)	0.30
2,4-DCP	Model 1	1.17(0.88,1.55)	0.27	1.03(0.73,1.45)	0.88
	Model 2	1.18(0.87,1.61)	0.24	1.07(0.78,1.47)	0.62
	Model 3	1.17(0.80,1.72)	0.32	1.13(0.73,1.74)	0.47
2,5-DCP	Model 1	1.08(0.92,1.25)	0.33	1.05(0.87,1.25)	0.61
	Model 2	1.09(0.92,1.29)	0.26	1.07(0.90,1.27)	0.41
	Model 3	1.08(0.88,1.33)	0.34	1.09(0.82,1.45)	0.43

*P < 0.05 was considered statistically significant.*

### 3.3 WQS analysis

**Figure 3:**
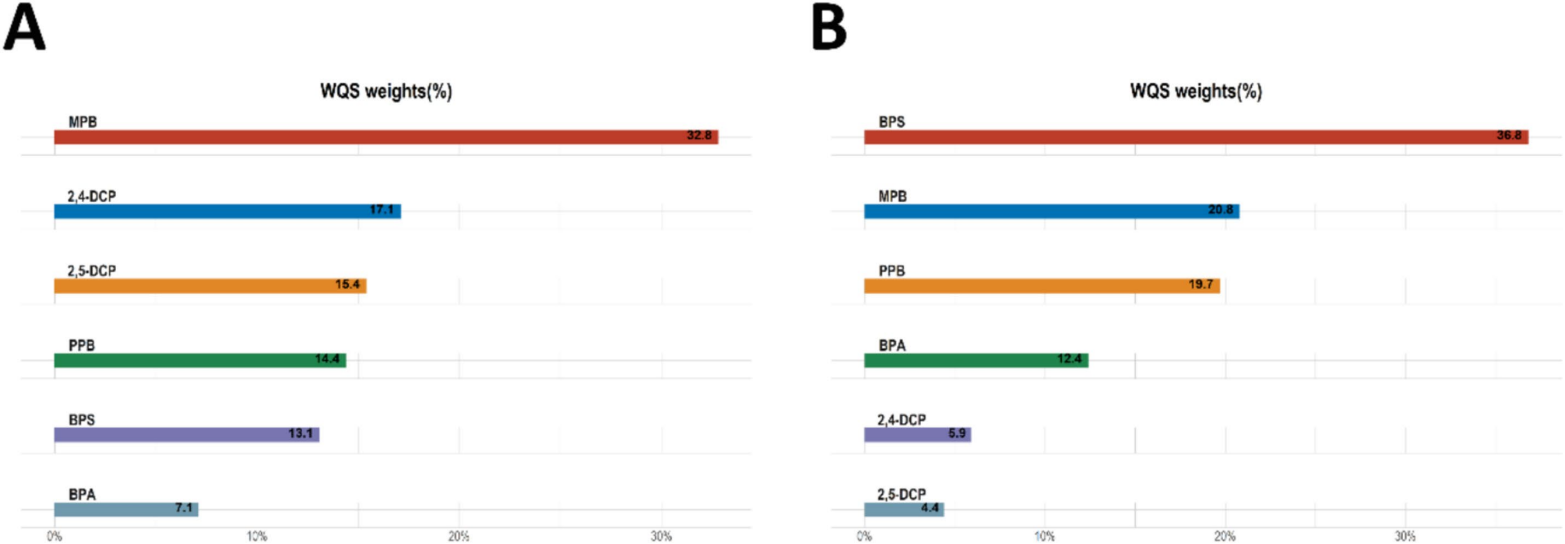
WQS analyses the importance of each chemical in the mixture for **(A)** cancer and **(B)** prostate cancer.

The results of the WQS analysis of the mixed effects of the six chemicals with cancers and prostate disease were shown in Table 4. The results of several sensitivity analyses showed that mixed compounds were positively associated with male cancers and prostate cancer. In the final adjusted model, each quartile increase in the WQS index was associated with a 78% (OR: 1.78, 95% CI: 1.10–2.87) increased risk of cancer and a 148% (OR: 2.48, 95% CI: 1.07–5.71) increased risk of prostate cancer. The importance of each chemical in the mixture was shown in Figure 3. The positive effect of MPB on cancer prevalence was weighted the most and BPA the least (Figure 3A). The positive effect of BPS on prostate cancer was weighted most heavily, with MPB and PPB similarly weighted more heavily. 2,4-DCP and 2,5-DCP were weighted less heavily (Figure 3B). No significant associations were found between WQS and cancer prevalence and prostate cancer prevalence in men when all *β* coefficients were assumed to be negative.

**Table 4:** Results of WQS analyses of the mixed effects of six chemicals and cancers and prostate disease.

Group	Outcomes	OR	95% CI	*p* value
Cancer	Model 1	1.80	1.18–2.74	0.01
	Model 2	1.79	1.12–2.87	0.02
	Model 3	1.78	1.10–2.87	0.02
Prostate	Model 1	1.97	1.07–3.64	0.03
	Model 2	1.99	1.04–3.80	0.04
	Model 3	2.48	1.07–5.71	0.03

### 3.4 Qgcomp analysis

**Figure 4:**
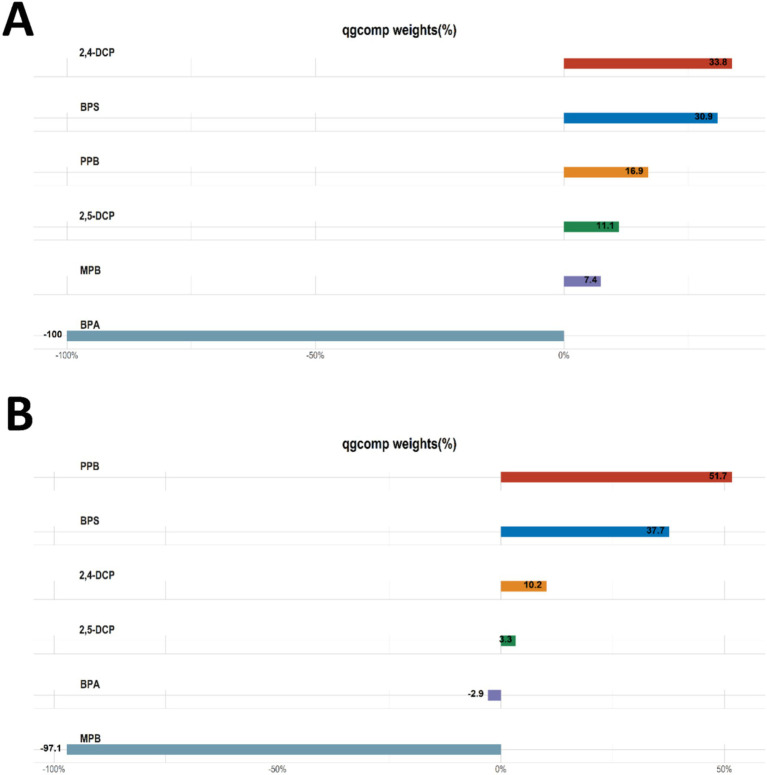
Qgcomp analyses the importance of each chemical in the mixture for **(A)** cancers and **(B)** prostate cancer.

The results of qgcomp analyses of the mixture of six chemicals with cancer and prostate disease were shown in Table 5. The results of several sensitivity analyses showed that chemical mixtures were positively associated with cancers and prostate cancer in men. In the final adjusted model, each quartile increase in the qgcomp index was associated with a 59% (OR: 1.59, 95% CI: 1.09–2.33) increase in the risk of cancer and a 105% (OR: 2.05, 95% CI: 1.04–4.06) increase in the risk of prostate cancer. The importance of each chemical in the mixture was shown in Figure 4. The effect weights of BPS, MPB, PPB, 2,4-DCP and 2,5-DCP on cancer prevalence were all positive, with BPS and 2,4-DCP having the largest effect weights and only BPA having a negative effect weight. BPS, PPB, 2,4-DCP and 2,5-DCP all had positive effect weights on prostate cancer. PPB had the largest effect weight of 51.7%. 2,5-DCP had the smallest effect weight. BPA and MPB had negative effect weights and MPB had a negative effect weight of 97.1%. It was worth noting that the results obtained from the WQS regression and the qgcomp regression were essentially the same.

**Table 5:** Results of qgcomp analyses of the mixed effects of six chemicals and cancers and prostate disease.

Group	Outcomes	OR	95% CI	*p* value
Cancer	Model 1	1.56	1.09–2.23	0.01
	Model 2	1.62	1.11–2.37	0.01
	Model 3	1.59	1.09–2.33	0.02
Prostate	Model 1	2.05	1.10–3.82	0.02
	Model 2	2.10	1.06–4.14	0.03
	Model 3	2.05	1.04–4.06	0.04

### 3.5 BKMR analysis

**Figure 5:**
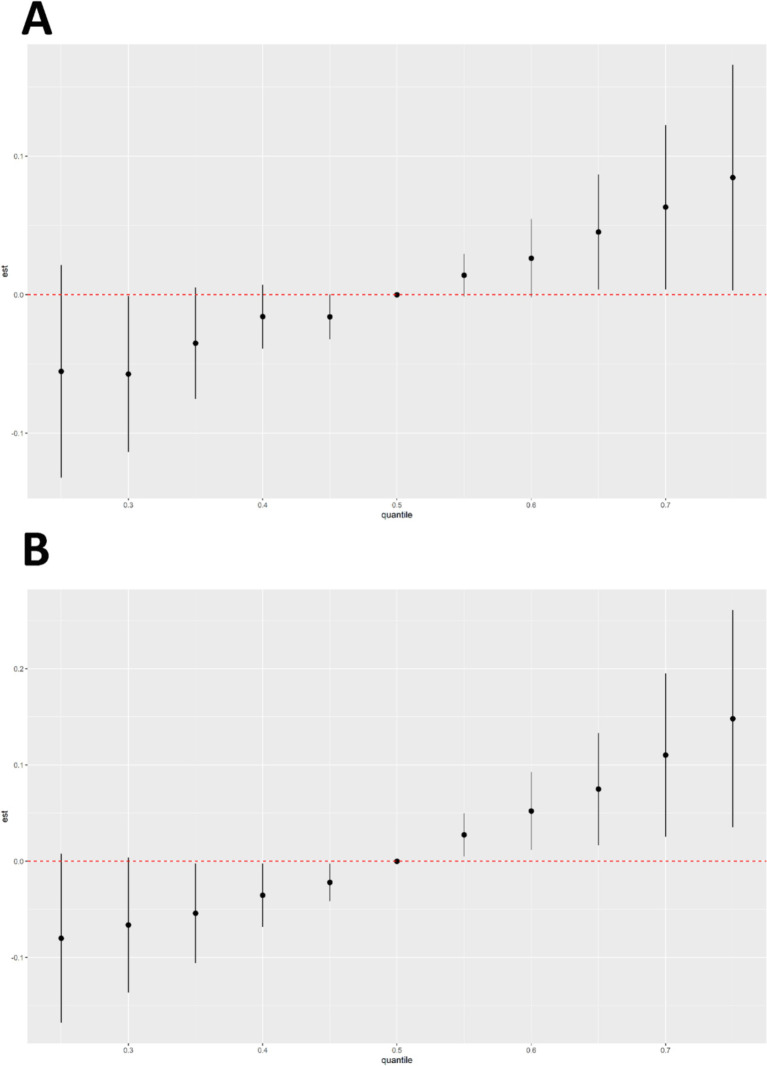
Results of BKMR analyses of six chemical mixtures in combination with male cancers **(A)** and prostate cancer disease **(B)**.

According to the results of BKMR model fitting, the combined effect of six chemical mixtures with male cancer and prostate cancer prevalence was shown in Figure 5. Chemicals at different levels were compared to the 50th percentile. When all chemicals were at the 65th percentile or higher, there was a gradual increase in the risk of cancer in men (Figure 5A). There was a progressive increase in the risk of prostate cancer in men when all chemicals were in the 55th percentile or higher (Figure 5B). The results showed a significant positive correlation between the chemical mixture and the two outcomes.

The three groups (groupPIP) and each chemical (condPIP) obtained from the BKMR model were shown in Table 6. The PIP for all groups in the male cancer prevalence analysis was less than 0.5, whereas in prostate cancer the PIP for all groups was greater than 0.5. The results indicated that all three groups are important drivers of joint action. In the phenol group, BPS had a condPIP of 0.69. In parabens, PPB had a condPIP of 0.74. Parabens was a significant contributor in both groups, and in the chlorophenol group the contributions of the two chemicals were almost identical.

**Table 6:** The three groups (groupPIP) and each chemical (condPIP) obtained in the BKMR model.

Chemicals	Group	Cancer		Prostate	
		groupPIPs	condPIPs	groupPIPs	condPIPs
BPA	1	0.11	0.08	0.61	0.31
BPS	1	0.11	0.92	0.61	0.69
MPB	2	0.09	0.92	0.78	0.26
PPB	2	0.09	0.08	0.78	0.74
2,4-DCP	3	0.31	0.99	0.58	0.52
2,5-DCP	3	0.31	0.01	0.58	0.48

**Figure 6:**
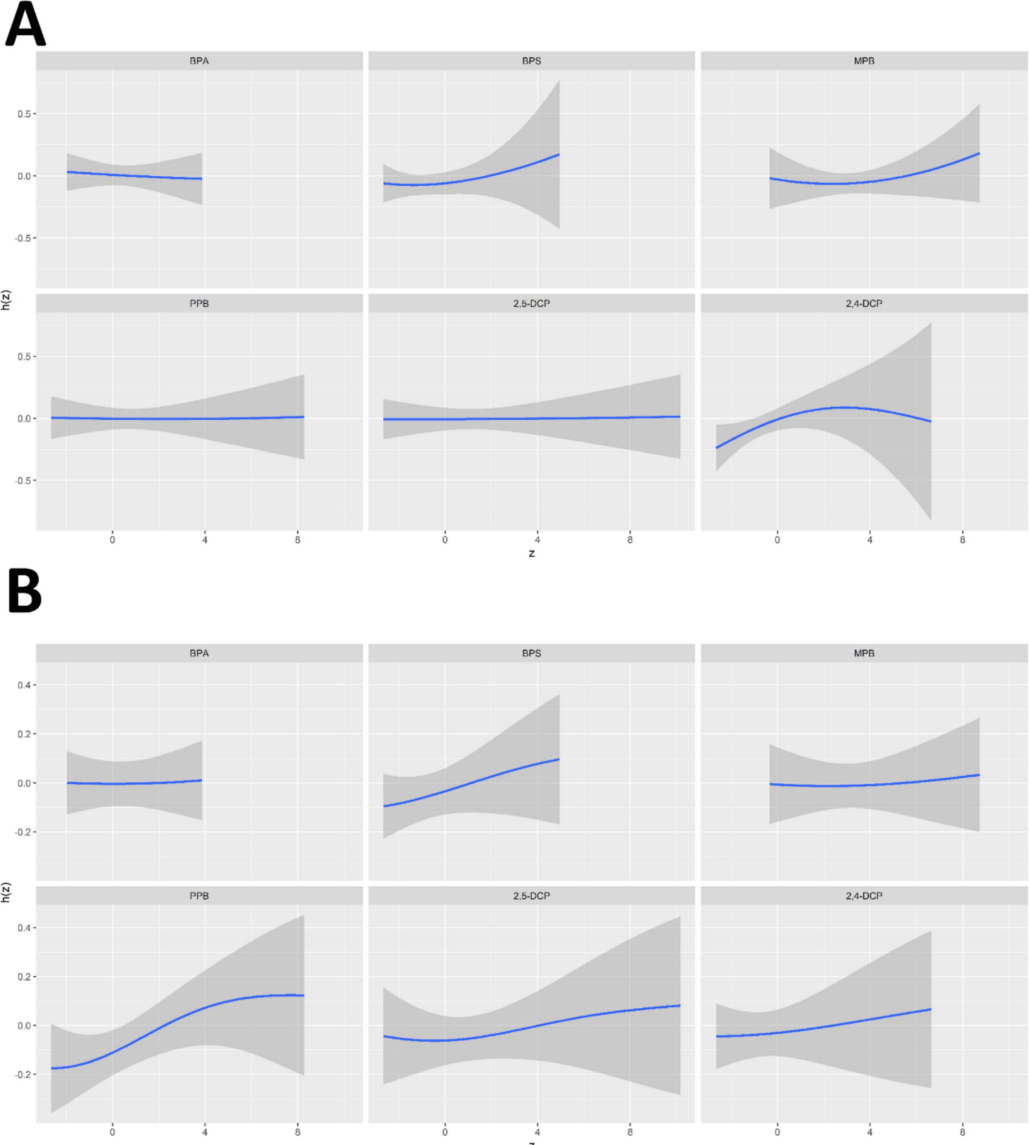
Univariate exposure-response relationships between six chemicals and the prevalence of **(A)** cancers and **(B)** prostate cancer in men.

The univariate exposure-response relationships between the six chemicals and the prevalence of cancer and prostate cancer in men were shown in Figure 6. 2,4-DCP was positively associated with cancer prevalence when all other chemicals were at median levels, with a slight decrease at the highest concentrations. BPS and MPB showed a positive correlation with cancer prevalence and BPA a negative correlation with cancer prevalence. PPB and 2,5-DCP showed a flat relationship (Figure 6A). The associations of BPS, PPB, 2,5-DCP, and 2,4-DCP with prostate cancer prevalence were monotonically increasing. The trend between MPB and prostate cancer prevalence was relatively flat in the first half of the trend and increasing in the second half of the trend. BPA showed a flat relationship with prostate cancer prevalence (Figure 6B).

**Figure 7:**
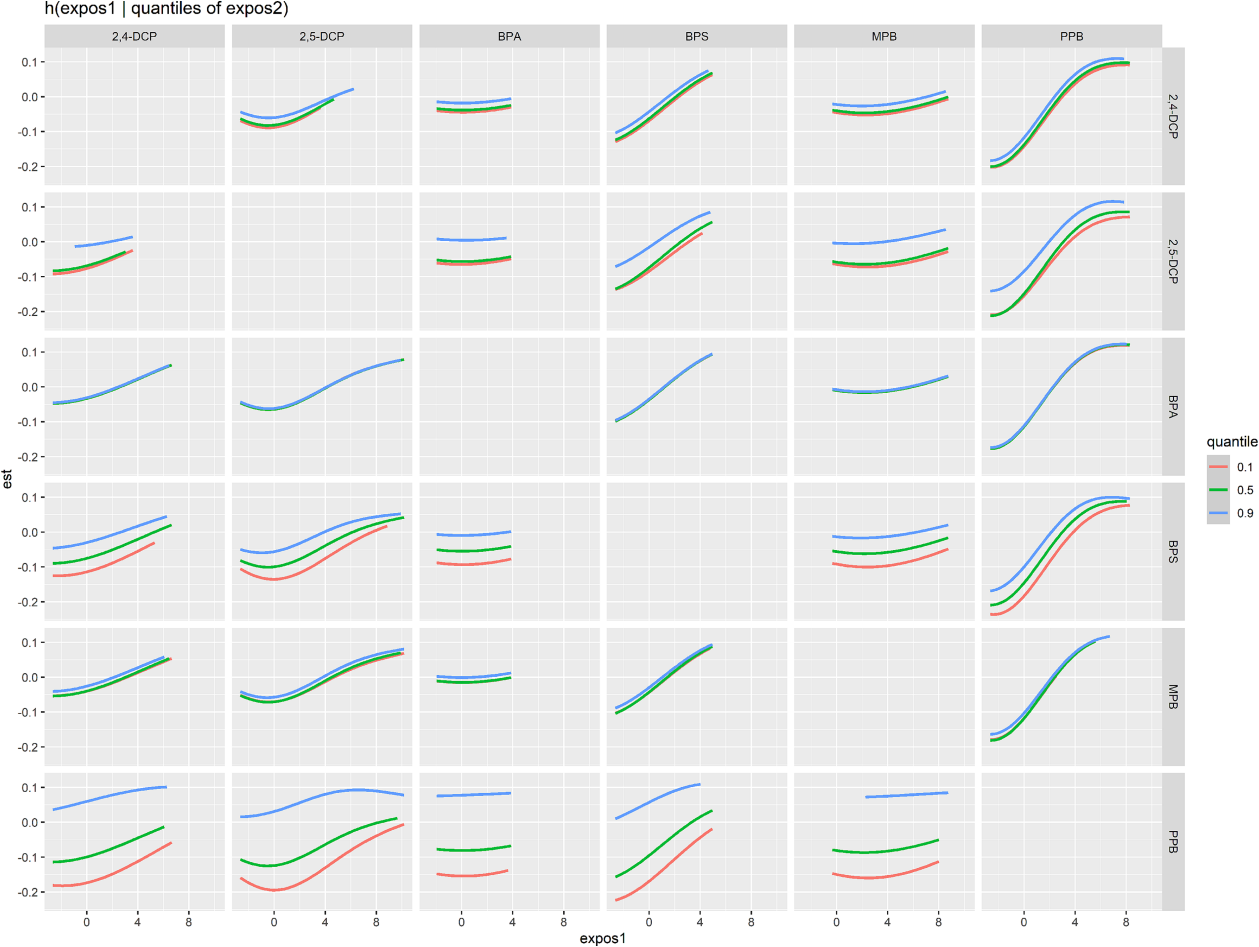
Bivariate exposure-response relationships for mixed chemicals.

The bivariate exposure-response relationship for a chemical substance was shown in Figure 7. The other chemicals were kept at the median level and the second chemical was taken at the 10th, 50th and 90th percentile to observe the possible interactions between the chemicals. The results showed that there may be potential interactions between 2,5-DCP, PPB, and MPB, and between BPS and 2,5-DCP.

**Figure 8:**
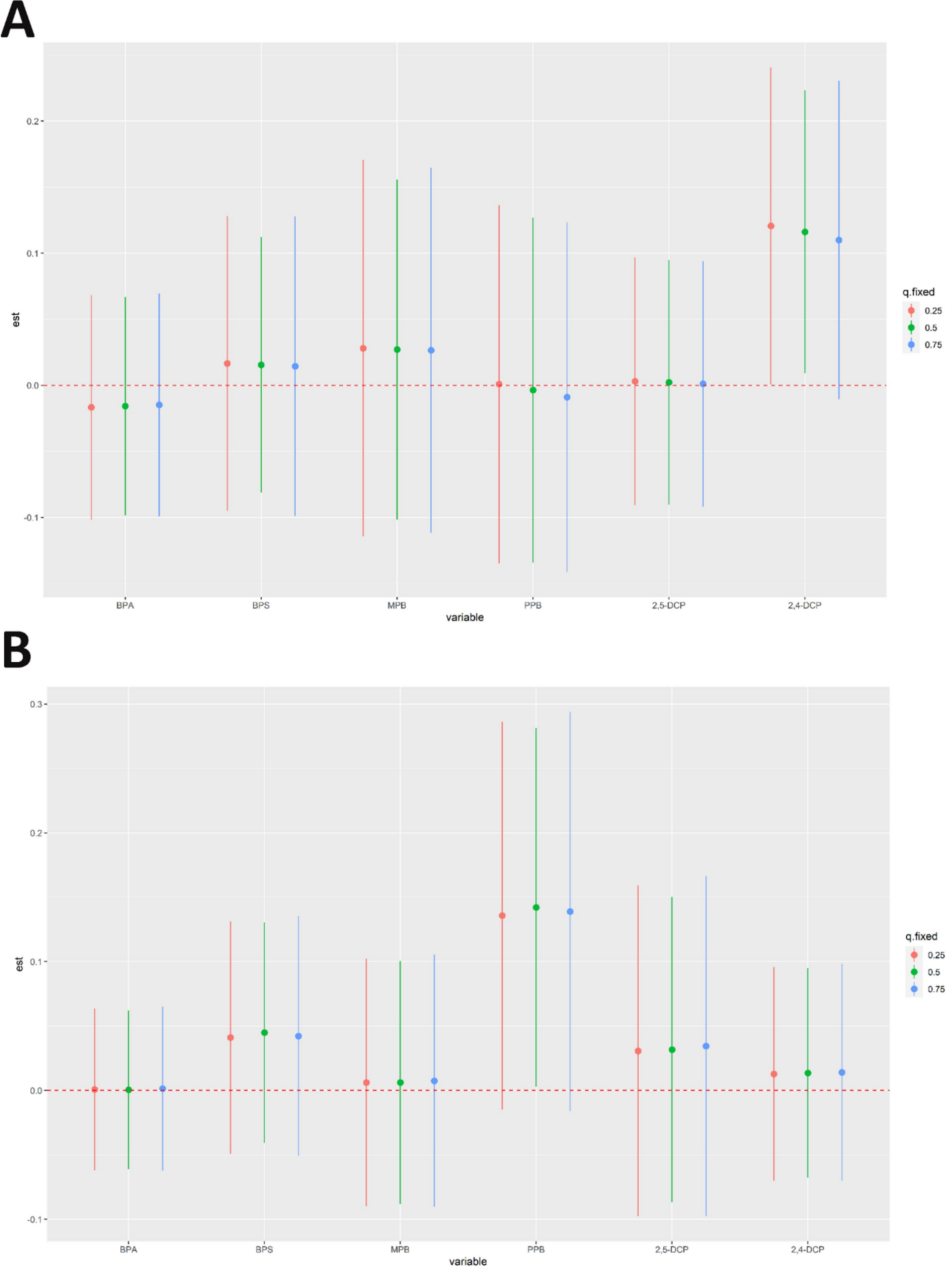
Results of analyses of the effects of individual chemicals on the prevalence of **(A)** cancers and **(B)** prostate cancer in men.

In addition, this study fixed the concentration of other metabolites at the 25th, 50th, and 75th quartiles to observe the effect of individual chemicals on the prevalence of cancer and prostate cancer in men. The results were shown in Figure 8. 2,4-DCP was positively associated with cancer prevalence when other chemical concentrations were in the 50th quartile (Figure 8A). PPB was positively associated with prostate cancer prevalence (Figure 8B).

### 3.6 RCS analysis

**Figure 9:**
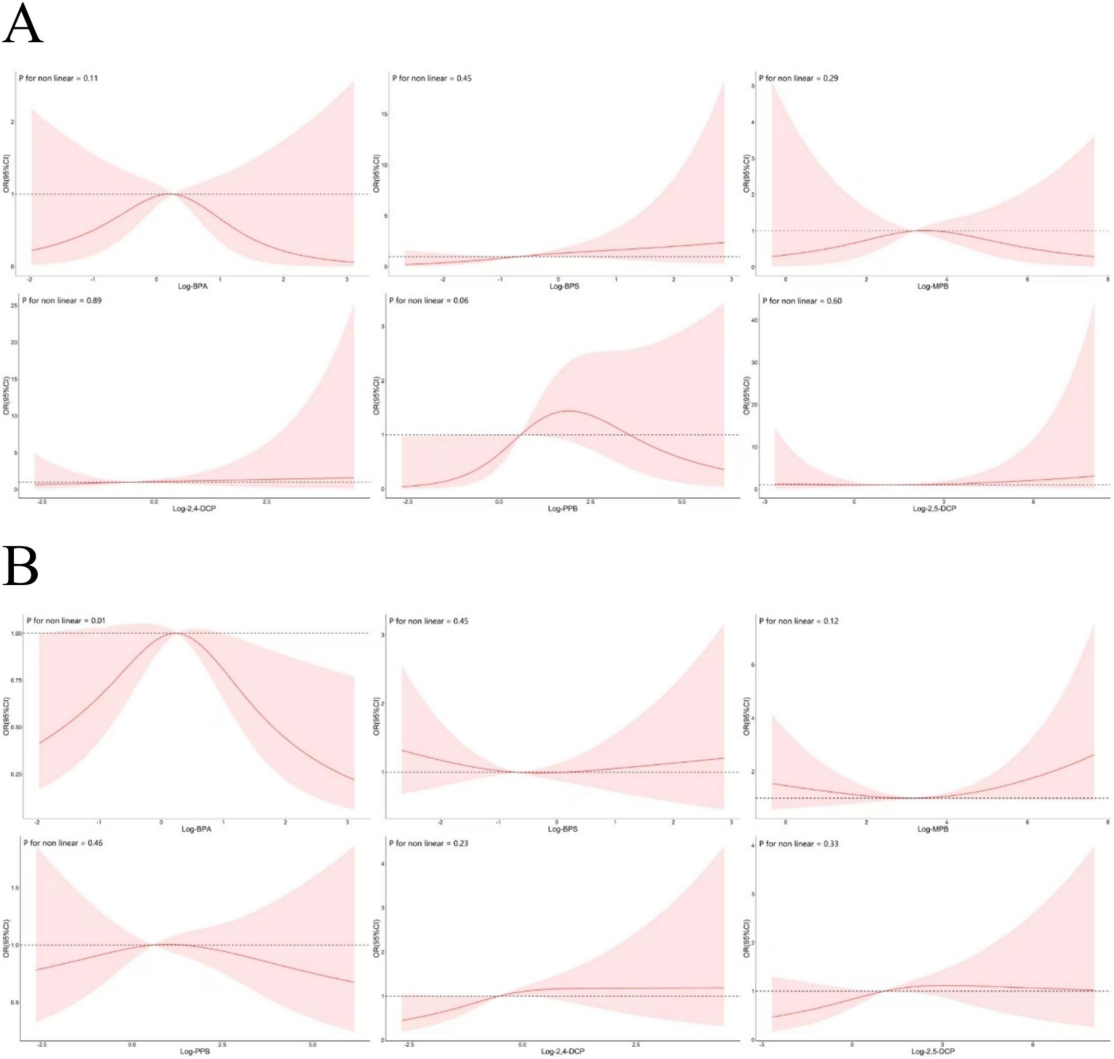
Non-linear associations between six chemicals and the prevalence of **(A)** cancers and **(B)** prostate cancer.

The adjusted RCS model described the non-linear relationship between the six chemicals and cancer risk was shown in Figure 9A. There was a significant non-linear relationship between BPA and cancer prevalence only (*p* = 0.01). There was a downward trend in the first half and an upward trend in the second half between BPS and MPB and cancer prevalence. In contrast to PPB, 2,4-DCP and 2,5-DCP were largely monotonically associated with cancer prevalence. The non-linear relationship with the risk of developing prostate cancer was shown in Figure 9B. There was an increasing trend in the first half and a decreasing trend in the second half between BPA, MPB, PPB and prostate cancer prevalence. BPS, 2,4-DCP and 2,5-DCP were largely monotonically associated with prostate cancer prevalence, and the results obtained by RCS were approximately the same as those of BKMR.

## 4 Discussion

PB is a preservative ingredient widely used in cosmetics, food, and pharmaceuticals. PB enters the human body through diet, inhalation, and skin contact (17). PB has attracted the attention of researchers for its endocrine-disrupting properties in humans. The physiological role of endocrine disruptors in chronic diseases and cancer is of increasing interest, and the detection of PB levels in urine is the most widely recognized biological sample (16). In animal model experiments, exposure to specific PBs has been associated with obesity, breast cancer, and prostate cancer (27–29). Relevant clinical studies have shown that PB has an impact on gene expression related to prostate cancer prognosis (16). In another study, MPB was found to upregulate the mRNA and protein expression levels and enzymatic activity of matrix metalloproteinase (MMP) in cancer cells (28). Notably, changes in MMP expression are critical for cancer progression. In some clinical studies on pathogenic mechanisms and therapeutic effects, it was found that changes in the expression of MMP have an important impact on cancer development, invasion, and migration (30, 31). The endocrine disruption and carcinogenicity of PB in humans have been demonstrated in a large number of *in vitro* and animal experiments, but the health effects in humans remain unclear (32). However, preventive measures for the effects of PB on human health are necessary. In a study on PB exposure and sperm motility in men, it was found that PB can lead to decreases in sperm concentration, number, and motility (33). Furthermore, studies have shown an association between PB exposure and cancer diagnosis (34). More importantly, PB has important effects on purine metabolism, lipid metabolism, and reactive oxygen (ROS) species metabolism (35–37).

BPA was included as an endocrine disruptor chemical. BPA is a chemical widely used in the manufacture of everyday consumer products. BPA has endocrine disrupting properties and is associated with many human diseases, including cancer (11). BPA, as a non-persistent chemical, is rapidly metabolized by the kidneys after ingestion into the body (38). Currently, an increasing number of bioassay studies are focusing on the levels of BPA and its analogs in urine (39). In a study on the relationship between bisphenolics and markers of oxidative stress, it was shown that elevated levels of bisphenolics in urine were associated with increased markers of oxidative stress. Numerous *in vitro* and animal experiments have shown that this correlation may be achieved through ROS release (8). There are no studies on the relationship between BPA and cancer prevalence in men. However, a positive correlation between BPA exposure and the risk of breast cancer has been noted (12). This relationship may suggest that bisphenols increase the risk of breast cancer by promoting estrogen-induced breast cancer or altering body weight (40). A strong correlation was found between levels of bisphenol exposure and the body composition of U.S. adults in a study of body composition. Bisphenols act to increase the risk of obesity and alter muscle fibers by disrupting the endocrine system (41). *In vivo* and *in vitro* studies have shown that BPS can promote adipocyte differentiation, induce adipogenesis, and interfere with lipid metabolism, among other processes (42).

As a chemical with endocrine-disrupting properties, DCP has a significant impact on human health (18). Studies have indicated that 2,5-DCP exposure can effectively induce glycolipid metabolism and lead to the development of metabolic syndrome (19). In addition, relevant studies have shown that DCP has an influential effect on ROS production *in vivo* (19, 43).

The six substances share three common characteristics: 1. Endocrine-disrupting features, especially lipid metabolism; 2. Association with ROS production; 3. High abundance in urine. Numerous previous studies have shown that the six chemicals have an effect on the body’s internal metabolism due to their inherent endocrine-disrupting properties. Evidence suggested that the above six chemicals were more important in vivo for their effects on lipid metabolism in the body (19, 35–37, 42). Studies have shown that dysregulation of lipid metabolism is one of the most prominent metabolic alterations in cancer significantly affecting cancer development, proliferation, invasion, metastasis, and treatment (44). Lipid metabolism plays an important role in the differentiation and value-added processes of cancer stem cells (45–48). Notably, lipid metabolism, as an essential part of cancer development, and disorders of lipid metabolism may be one of the reasons why the above six chemicals contribute to the development of cancer in men. In addition, it is well known that disorders of lipid metabolism are closely associated with the prostate (49–51). Lipid metabolism related factors such as BMI, waist circumference and diabetes mellitus were used as variables for statistical analysis. The results showed that the association between the six chemicals and cancer prevalence remained unchanged after adjusting for lipid metabolism-related variables. Therefore, it is hypothesized that these six chemicals may influence the development of cancer in men by affecting lipid metabolism in the body.

ROS are highly reactive substances containing oxygen free radicals that act as tumor suppressors or tumor promoters. Studies have shown that most chemotherapeutic agents promote apoptosis of tumor cells by generating ROS. However, higher levels of ROS may trigger tumourigenesis (52). Studies have pointed out that high levels of metabolically produced ROS are one of the important markers of cancer (53). As a substance with a dual role in cancer, metabolic alterations of ROS have an important impact on cancer development and suppression (54). Notably, a large number of studies have shown that the six chemicals have an effect on ROS metabolism (8, 37, 43). As a highly biologically active molecule, ROS have been extensively studied, especially for the treatment of various cancers and the mechanism of carcinogenesis (55, 56). A large number of studies have now shown that prostate occurrence is associated with increased ROS (57, 58). Previous studies have shown that the six chemicals have an important role in influencing ROS production *in vivo*. However, further experimental validation is needed to explore the mechanisms involved.

We observed a significant increase in cancer risk when chemical concentrations exceeded the 65th percentile. At low concentrations, cells may repair damage caused by chemicals through repair mechanisms. However, at high concentrations, the accumulation of damage may exceed the cell’s repair capacity, thereby increasing cancer risk (59). We observed decrease in cancer risk at high concentrations of 2,4-DCP, which indeed suggests a nonlinear relationship. We hypothesize that this may be related to the following mechanisms: 1. Cellular Adaptive Responses: At high concentrations, cells may activate adaptive responses (e.g., antioxidant defenses, enhanced DNA repair, or apoptosis), thereby reducing carcinogenic risk. This phenomenon is known as the “hormesis effect” in toxicology (60). 2. Cytotoxicity-Induced Cell Death: Extremely high concentrations of 2,4-DCP may directly induce cell death (e.g., apoptosis or necrosis), thereby reducing the survival of potentially cancerous cells (61). For the nonlinear relationship between BPA and cancer risk, we propose: 1. Receptor Saturation Effect: As an endocrine-disrupting chemical, bisphenol A may exert its effects by binding to estrogen receptors (ER). At low concentrations, bisphenol A may mimic estrogenic effects and promote cell proliferation; however, at high concentrations, receptor saturation may occur, leading to weakened or altered effects (62). 2. Metabolic pathway shifts: High concentrations of bisphenol A may activate different metabolic pathways, resulting in reduced generation or increased clearance of its toxic metabolites (63).

Urine, as a dynamic excretion with variable properties, is generally recognized as a better source of biomarkers than blood (64). In the demographic characterisation study of this study, MPB was found to be the most abundant of the 6 chemicals. The results of the WQS regression analyses showed that MPB had the highest weight of positive effect on cancer prevalence in men, and also occupied a high position in the weight of effect for prostate cancer. The weight of the role of MPB was as high as 97.1%. It is believable that the higher the level of PB exposure, the higher the risk of cancer prevalence in men, especially prostate cancer. However, the esoteric mechanisms need to be further explored and discovered. Considering the endocrine-disrupting and ROS-producing effects of the six chemicals, it is reasonable to believe that these chemicals promote cancer in men by inducing disruption of lipid metabolism and ROS production. Therefore, more longitudinal studies as well as animal studies are necessary to validate the analyze. Based on the study’s findings that BPS, PPB, and 2,4-DCP are associated with an increased risk of cancer in men, we propose the following public health recommendations: 1. Reduce usage. Relevant authorities should evaluate and limit the use of BPS, PPB, and 2,4-DCP in consumer products (e.g., plastics, cosmetics, and cleaning agents), particularly in food packaging and personal care items. 2. Promote alternatives. Encourage the development and adoption of safer chemical alternatives to minimize potential health risks associated with these substances. 3. Strengthen regulation. Implement stricter safety standards and enhance regulatory oversight of BPS, PPB, and 2,4-DCP levels in food, cosmetics, and other consumer products, with regular monitoring of their usage. 4. Public awareness campaigns: Increase public awareness of the potential health risks posed by these chemicals and promote the selection of products free from these substances to reduce exposure risks.

The following limitations exist in this study: 1. Limited causality. The cross-sectional design results in simultaneous measurement of exposure and outcome variables, making it difficult to specify time-series relationships. Behavioral changes in patients after cancer diagnosis (e.g., medication use or dietary modifications) may inversely affect urinary biomarker levels, with a risk of reverse causation. 2. Unmeasured confounders. Despite adjusting for multidimensional covariates, complex factors such as history of occupational exposure, genetic susceptibility, and family history may be incompletely captured or may affect the accuracy of exposure-outcome associations. 3. Transient biomarkers. A single urine test is difficult to characterize long-term exposure to non-persistent chemicals, intra-individual biological variation may introduce measurement error, and repeated sampling is needed to improve the reliability of exposure assessment. 4. Outcome misclassification. Reliance on self-reported cancer diagnostic data may produce classification bias, especially for non-prostate cancer types with high rates of misdiagnosis, and the lack of pathologic validation may undermine the credibility of the results. 5. Exposure window limitations. Urine indicators mainly characterize short-term metabolic exposures and are not effective in assessing chronic cumulative effects of chemicals, and need to be combined with data on persistent pollutants in blood / tissue samples to improve the exposure assessment system.

## 5 Conclusion

In this cross-sectional study of nationally representative urine samples from men in the United States, levels of mixed chemicals in urine were positively associated with the prevalence of cancers in men. This correlation also exists for prostate cancer. This finding has important clinical implications for public health, particularly for the prevention and early screening of male cancers, especially prostate cancer. A deeper understanding of the pathogenic mechanisms of the six chemicals in this study through more in-depth research explorations could help to specify more effective interventions. With a deeper knowledge of the mechanisms, perhaps biomarkers in urine will play an even more important role, not just in male cancers.

## Data Availability

The raw data supporting the conclusions of this article will be made available by the authors, without undue reservation.

## Footnotes

^1^ https://wwwn.cdc.gov/nchs/data/nhanes/2013-2014/labmethods/EPHPP_H_MET
